# Research on the multidimensional brain remodeling mechanisms at the level of brain regions, circuits, and networks in patients with chronic lower back pain caused by lumbar disk herniation

**DOI:** 10.3389/fnins.2024.1357269

**Published:** 2024-03-06

**Authors:** Yuan-Dong Mei, Hang Gao, Wei-Fei Chen, Wei Zhu, Chen Gu, Jun-Peng Zhang, Ji-Ming Tao, Xu-Yun Hua

**Affiliations:** ^1^Department of Hand Surgery, the Second People’s Hospital of Changshu, Changshu, China; ^2^Department of Rehabilitation, Shuguang Hospital Affiliated to Shanghai University of Traditional Chinese Medicine, Shanghai, China; ^3^School of Rehabilitation Science, Shanghai University of Traditional Chinese Medicine, Shanghai, China; ^4^Department of Traumatology and Orthopedics, Yueyang Hospital of Integrated Traditional Chinese and Western Medicine, Shanghai University of Traditional Chinese Medicine, Shanghai, China

**Keywords:** chronic lower back pain, lumbar disk herniation, functional magnetic resonance imaging, functional connectivity, network

## Abstract

**Introduction:**

Chronic lower back pain (cLBP), frequently attributed to lumbar disk herniation (LDH), imposes substantial limitations on daily activities. Despite its prevalence, the neural mechanisms underlying lower back pain remain incompletely elucidated. Functional magnetic resonance imaging (fMRI) emerges as a non-invasive modality extensively employed for investigating neuroplastic changes in neuroscience. In this study, task-based and resting-state fMRI methodologies are employed to probe the central mechanisms of lower back pain.

**Methods:**

The study included 71 chronic lower back pain patients (cLBP group) due to LDH and 80 age, gender, and education-matched healthy volunteers (HC group). The subjects are mainly middle-aged and elderly individuals. Visual Analog Scale (VAS), Oswestry Disability Index (ODI), and Japanese Orthopedic Association Scores (JOA) were recorded. Resting-state and task-based fMRI data were collected.

**Results/discussion:**

No significant differences were observed in age, gender, and education level between the two groups. In the cLBP group during task execution, there was diffuse and reduced activation observed in the primary motor cortex and supplementary motor area. Additionally, during resting states, notable changes were detected in brain regions, particularly in the frontal lobe, primary sensory area, primary motor cortex, precuneus, and caudate nucleus, accompanied by alterations in Amplitude of Low Frequency Fluctuation, Regional Homogeneity, Degree Centrality, and functional connectivity. These findings suggest that chronic lower back pain may entail reduced excitability in sensory-motor areas during tasks and heightened activity in the sensory-motor network during resting states, along with modified functional connectivity in various brain regions.

## Introduction

Chronic low back pain (cLBP) typically denotes enduring discomfort and pain in the back or lumbar region persisting for over 3 months, frequently accompanied by radiating pain in the lower limbs (Zhou et al., 2019; Thompson et al., 2020). This condition not only profoundly impacts individuals’ lives but also imposes a burden on society and the economy. It is a primary cause of restricted activity, work-related loss, and other associated disabilities (Disease, G.B.D., Injury, I., and Prevalence, C et al., 2016, 2017; Foster et al., 2018; Hartvigsen et al., 2018). With the aging population and global demographic changes, the severity of this issue is progressively increasing (Clark and Horton, 2018). Reported prevalence rates of cLBP vary across different age groups: it is around 4.2% among individuals aged 20–30, and approximately 24.8% among those aged 50 and above (Jackson et al., 2015; Iizuka et al., 2017). According to researchers’ estimates in 2020, the global prevalence of low back pain (LBP) is 7.5%, affecting approximately 577 million people (Jahn et al., 2023). Although LBP can be temporary with a fluctuating pattern of recovery, it is estimated that 4–20% of the adult population may develop a chronic condition that gradually increases with age. Meanwhile, the number of individuals globally disabled due to cLBP is also rising year by year (Hoy et al., 2012). In China, between 1990 and 2016, although the prevalence of lower back pain has slightly decreased, the total number of affected individuals and years lived with disability have increased (Wu et al., 2019).

Lumbar disk herniation (LDH) is a syndrome resulting from the degeneration of intervertebral disks and the subsequent rupture of the fibrous ring, leading to the protrusion of the nucleus pulposus. This protrusion can stimulate or compress nerve roots and the cauda equina (Rossi et al., 2004). The earliest and most common symptom is typically lower back pain (Kanna et al., 2014). Most LDHs occur at the L4-5 and L5-S1 levels, often leading to sciatica (Bailey et al., 2020; Lee et al., 2020). Higher-level herniations can compress the LI, L2, and L3 nerve roots, causing pain in the groin or inner thigh. Nearly 80% of the population experiences LBP at least once in their lifetime, with LDH being the most common cause (Amin et al., 2017). LDH refers to a condition where the fibrous ring ruptures due to various factors, causing the nucleus pulposus to protrude and exert varying degrees of pressure and stimulation on nerve roots, resulting in a series of clinical symptoms and signs (Postacchini and Postacchini, 2011). Posterior protrusion of disk tissue can compress the cauda equina, resulting in cauda equina syndrome, characterized by saddle area sensory abnormalities, acute urinary retention, and loss of bowel control (Chen et al., 2020; Grasso et al., 2020; Lee et al., 2020). Some patients with lumbar disk herniation may not experience leg pain but instead exhibit numbness in the limbs, which is due to stimulation of proprioceptive and tactile fibers by the protruding disk tissue.

Currently, the treatment of cLBP often proves to be unsatisfactory. Over the past 50 years, the use of painkillers has not yielded favorable results, and there has been limited development of new non-opioid and non-addictive pain medications (Kim et al., 2019). This suggests a pressing need to better understand the pathophysiological mechanisms of the disease and formulate new treatment approaches. Moreover, cLBP is influenced by a range of factors including biological, physiological, psychological, and social elements, leading to significant variations in its etiology (Chen et al., 2018).

Furthermore, cLBP is intertwined with physiological and psychological factors. Many patients seeking treatment exhibit symptoms of mental and physical disorders like depression and anxiety, which negatively impact treatment outcomes (Martucci and Mackey, 2018). While imaging can explain simple mechanical compression mechanisms like fibrous ring rupture and nucleus pulposus protrusion, it cannot entirely elucidate how the clinical presentation of cLBP patients affects psychosomatic symptoms. Increasing severity and duration of pain might lead to emotional changes such as anxiety and depression (Kong et al., 2013; Yu et al., 2014; Zhang et al., 2019), which can, in turn, exacerbate pain. Moreover, prolonged use of painkillers by cLBP patients can lead to dependency in some cases, further contributing to psychosomatic harm. However, some clinicians still adhere to a traditional understanding of the disease, focusing solely on localized physical changes, without fully recognizing the close relationship between psychosomatic symptoms such as depression and anxiety caused by cLBP and changes in brain function.

Indeed, the neuropathological mechanisms underlying cLBP are not fully elucidated, particularly the relationship between brain functional changes and clinical symptoms. Clinical studies suggest that individuals with long-term chronic pain may gradually develop cumulative brain damage due to repeated pain-related processes, accompanied by impairments in sensory, cognitive, memory, and emotional functions. Previous neuroimaging research has indicated that the central nervous system is involved in the development, maintenance, and exacerbation of chronic pain (Martucci and Mackey, 2018), while also revealing associations between cLBP and changes in brain structure and physiological functions (Kong et al., 2013; Yu et al., 2014; Yang et al., 2017; Li et al., 2018; Zhang et al., 2019). Furthermore, research has found a link between cLBP and neurodegenerative changes in brain structure, which might accelerate brain aging and consequently impact cognitive function (Yu et al., 2021). However, the exact mechanisms and complex interactions between pain perception, brain changes, and functional deficits are still areas of active investigation.

Resting-state functional magnetic resonance imaging (rs-fMRI) is a non-invasive brain functional detection technique that provides important information for understanding disease mechanisms and guiding clinical practice. This technique has garnered significant attention from clinicians and researchers due to its numerous advantages, including non-ionizing radiation, high image clarity, ease of localization, and repeatability (Yu et al., 2014; Letzen and Robinson, 2017; Yang et al., 2017; Goossens et al., 2018; Li et al., 2018; Huang et al., 2019; Yu et al., 2021; Tang et al., 2023), and it does not require subjects to perform specific tasks. The brains of chronic pain patients need to continuously process spontaneous pain, which might interfere with other conscious and unconscious processes (Borsook et al., 2018). Furthermore, chronic pain can disrupt the flow of information and integration between brain regions, consequently impacting brain function and structure. Therefore, by comparing brain activity and network differences between patient groups and healthy controls, rs-fMRI provides valuable insights into the neural mechanisms underlying chronic pain. Moreover, task-related fMRI is utilized to investigate the correlation between brain activity and the execution of specific cognitive tasks, thereby enhancing our understanding of task localization within the brain.

### Overview of this study

This study aims to investigate the central mechanisms underlying lower back pain in patients using task-based and resting-state fMRI. Specifically, it seeks to elucidate the multidimensional remodeling of brain function and structure resulting from nerve root compression caused by LDH, with a particular focus on the voxel-circuit-network level.

## Materials and methods

### Participants

A total of 71 patients diagnosed with cLBP due to LDH, who received treatment at Changshu Second People’s Hospital from June 2021 to April 2023, were selected as the study subjects (cLBP group). Simultaneously, 80 age, gender, and education level-matched healthy volunteers were recruited from nearby communities and among patient family members as the normal control group (HC group). All participants were well-informed about the study’s content and potential discomforts or risks during the research process and voluntarily signed informed consent forms. cLBP group inclusion criteria: (1) confirmed lumbar disk herniation through clinical presentation, signs, and lumbar CT/MRI imaging, with chronic lower back pain as the main symptom and mild lower limb pain or numbness; (2) pain duration exceeding 3 months, either persistently or intermittently; (3) all patients underwent a pain visual analog scale (VAS) assessment before examination, with a score > 3 but tolerable (<8); (4) absence of mental disorders and other chronic pain conditions; (5) right-handed individuals; (6) capable of understanding and independently completing questionnaires, and able to abstain from pain medication for 7 days before the examination. cLBP group exclusion criteria: (1) individuals unable to cooperate due to severe pain for fMRI scanning or those with contraindications for MRI; (2) those with other chronic pain conditions; (3) participants with head motion >3 mm or rotation >3° during fMRI examination; (4) individuals with abnormal brain lesions identified through routine MRI; (5) pregnant or lactating individuals. Normal control group inclusion criteria: (1) no history of chronic pain or serious health issues; (2) no consumption of pain medication in the past 7 days; (3) right-handed individuals; (4) age, gender, and education level matching the cLBP group. Normal control group exclusion criteria: Same as the cLBP group.

### Clinical assessment

General demographic data collection, including participant ID, name, gender, age, handedness, ethnicity, marital status, education level, enrollment date, home address, and contact number, is recorded using a self-made case booklet. Additionally, medical history is gathered for chronic lower back pain patients, encompassing age of onset, duration of pain (in months), history of previous medication treatment, and surgical history.

### Functional assessment

The Visual Analog Scale (VAS) serves as the primary indicator in this study, accompanied by the Oswestry Disability Index (ODI) and Japanese Orthopedic Association Scores (JOA), alongside functional magnetic resonance imaging indicators, as secondary measures. The VAS, ranging from 0 to 10, assesses pain levels in lower back pain patients, with criteria as follows: 0–3 points for slight pain, 4–6 points for tolerable pain affecting sleep, and 7–10 points for intense pain. The ODI evaluates functional impairment through 10 questions on daily activities, scored from 0 to 5, with higher scores indicating more severe impairment. The JOA, ranging from 0 to 29, assesses treatment effectiveness based on patients’ symptoms, clinical signs, daily activities, and bladder function, with lower scores indicating greater functional impairment.

### Functional magnetic resonance assessment

Data collection: All data were collected using a Siemens MAGNETOM Verio 3.0 T MRI scanner from Germany. During the scanning process, patients were in a supine position, with their eyes closed and head immobilized. They wore noise-canceling earplugs, maintained calm breathing, remained awake, and minimized body movement.

EPI scan parameters: Repetition time (TR) = 3,000 ms; Echo time (TE) = 30 ms; Flip angle = 90°; Slice thickness = 3 mm; Number of slices = 43; Matrix size = 64 × 64; Field of view (FOV) = 230 mm × 230 mm; Voxel size = 3.6 mm × 3.6 mm × 3 mm; Total of 200 time points were collected.

T1 sequence scan parameters: TR = 1900 ms; Inversion time = 900 ms; TE = 2.93 ms; Flip angle = 9°; FOV = 256 mm × 256 mm; Slice thickness = 1 mm; Matrix size = 256 × 256.

### Data preprocessing

During the scanning process, physiological signals (such as respiration and heartbeat), involuntary minor head movements, and scanner performance status can introduce interference to fMRI scans, reducing accuracy. To minimize uncontrollable interference factors and improve data quality, data collected needs to undergo preprocessing. MATLAB (R2013b; The MathWorks, Natick, MA) processing platform and the SPM12 toolbox^1^ were used for preprocessing.

Functional image data preprocessing: (1) removal of the first 10 time points: at the start of the scan, there might be non-uniform effects in the magnetic field, and participants may undergo adaptive adjustments. To eliminate these interferences, data from the initial 10 time points are removed; (2) temporal slice correction: fMRI scans are acquired slice by slice, and during the whole-brain scan, there can be temporal discrepancies between slices due to differences in scanning time. Temporal slice correction is performed to eliminate differences caused by these temporal discrepancies; (3) head motion correction: due to the relatively long duration of fMRI scans, participants may not keep their heads entirely still throughout the scan. Involuntary head motion can significantly affect fMRI data. Thus, the sequence images acquired at different times are aligned and adjusted to the first frame image (with translations <2 mm or rotations <2°); (4) spatial normalization: because individual differences exist in brain morphology, participants’ brain images need to be registered to a standard template (in this case, an echo-planar imaging template) to achieve spatial normalization of all individual brain images and improve data quality; (5) Gaussian smoothing: to increase signal-to-noise ratio and ensure the fulfillment of the data’s random Gaussian field nature, Gaussian smoothing is applied to the data. The smoothing kernel size is generally set to twice the voxel size. If performing local consistency data analysis, Gaussian smoothing is often applied as the final step; (6) removal of linear drift: to eliminate system noise caused by scanner instability, including baseline drift and signal fluctuations; (7) bandpass filtering: to remove physiological information such as respiration, heartbeat, and involuntary movements causing whole-body muscle movements.

T1 image data preprocessing: (1) tissue segmentation: gray matter, white matter, and cerebrospinal fluid are segmented, resulting in a gray matter structure density map; (2) spatial registration: nonlinear segmentation registration (diffeomorphic anatomical registration through exponentiated lie algebra, DARTEL) combined with affine transformation is used to register the segmented individual brain gray matter density map to the MNI standard space template, and it is resampled to a voxel size of 1.5 × 1.5 × 1.5 mm^3^. The gray matter density map is then multiplied by the nonlinear deformation parameters obtained from the spatial registration process, resulting in a modulated gray matter probability map, known as the gray matter volume (GMV) map; (3) spatial smoothing: this step enhances signal-to-noise ratio and compensates for deviations introduced during spatial registration.

### Calculation of fMRI metrics

Task activation: a general linear model is used to statistically analyze all pixel points in each image. Parameters are estimated and family wise error (FWE) correction is applied. Brain activation areas are superimposed onto standardized structural images, and coordinates and activation area size are located and assessed using an automated anatomical labeling (AAL) toolbox.

Amplitude of low frequency fluctuation (ALFF): ALFF represents the strength of spontaneous activity for each voxel. It calculates the average of all frequency points within a frequency band (0.01–0.08 Hz), transforms the time series into a frequency range using Fourier transform, computes the square root of the power spectrum for each frequency, and the average of these square roots is the ALFF value. ALFF reflects the brain’s spontaneous activity during resting state from an energy perspective.

Regional homogeneity (ReHo): ReHo assumes that a voxel in a brain area correlates highly with its 26 neighboring voxels in the time series. Kendall’s coefficient of concordance (KCC) is computed to evaluate the synchronization of temporal changes within a cluster of 27 voxels. ReHo values for each participant are calculated using REST software (Song et al., 2011). ReHo values represent the KCC value of a selected voxel within the chosen area, ranging from 0 to 1. Values closer to 1 indicate better consistency, while values away from 1 indicate lower consistency.

Degree centrality (DC): DC is the most direct metric used to characterize the centrality of nodes in network analysis. It measures how many neighboring nodes a node has, i.e., the number of nodes directly connected to it. The larger the degree of a node, the higher its degree centrality, indicating greater importance of that node within the network.

Functional connectivity (FC): FC refers to the correlation of time series between spatially distinct brain regions. It assumes that neurons in a specific brain area influence neurons in other areas through outgoing pathways. This can be validated by correlational analysis of the time series of these two regions. If signals between regions exhibit high consistency, it suggests they constitute a closely related network.

### Statistical analysis

General information and clinical scale scores of participants are analyzed using SPSS 25.0 software. Chi-square test is used to compare gender differences between groups, and independent sample t-test is used to compare age, disease duration, and years of education differences between groups. The mean time series of ALFF and FC values from significantly different brain regions in the cLBP group are extracted. Pearson correlation analysis is performed between these time series and clinical scale scores. The significance level is set at *p* < 0.05 to indicate statistically significant differences.

## Results

### Demographic data

A total of 71 cLBP patients were included (39 males / 32 females), along with 80 healthy volunteers (42 males /38 females). There were no statistically significant differences between the two groups in terms of age, gender, and education level. Refer to Table 1 for details.

**Table 1:** Population demographics of chronic lower back pain group and normal group.

	Chronic lower back pain (*n* = 71)	Normal (*n* = 80)	t/χ^2^	*p*
Age	49.31 ± 9.13	47.68 ± 8.43	1.891	0.083
Gender	39/32	42/38	0.089	0.765
Years of education	13.89 ± 3.15	14.72	0.357	0.349

### Clinical scale data

Chronic low back pain patients had a relatively long duration of illness, with an average of 1.7 ± 0.45 years. The average pain intensity measured by VAS was 6.41 ± 2.47. The average ODI functional disability score was 44.65 ± 9.11, and the average JOA score was 12.24 ± 2.47. Refer to Table 2 for details.

**Table 2:** Clinical data.

	Chronic lower back pain
Years of cLBP	1.7 ± 0.45
VAS	6.41 ± 2.47
ODI	44.65 ± 9.11
JOA	12.24 ± 2.47

cLBP, Chronic Lower Back Pain; VAS, visual analog scale; ODI, Oswestry Disability Index; JOA, Japanese Orthopedic Association Scores.

### Task-based functional MRI results

**Figure 1:**
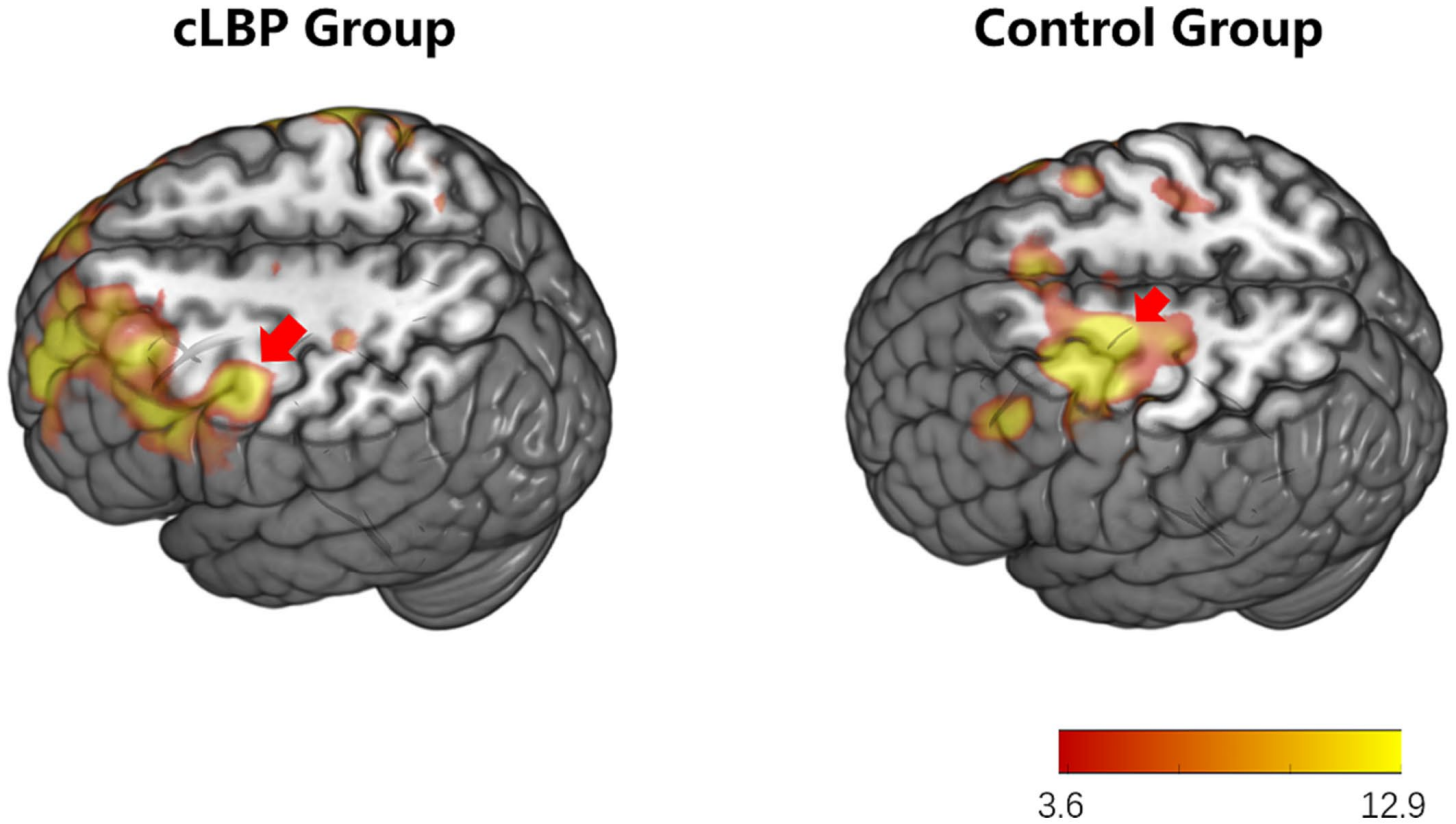
Brain activation maps during limb movement in chronic lower back pain patients and normal controls. The areas indicated by the red arrows correspond to the primary motor cortex and supplementary motor area.

**Figure 2:**
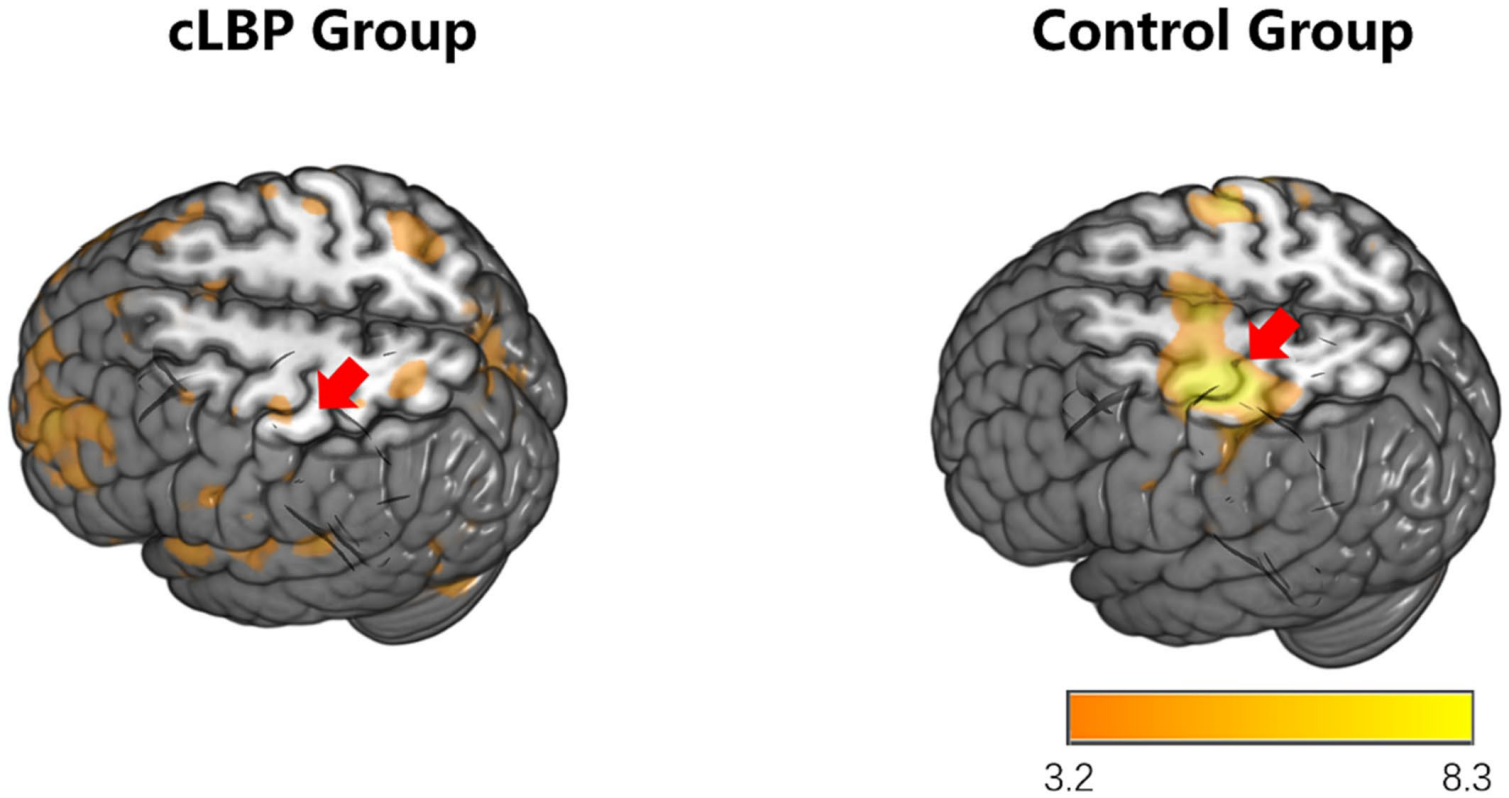
Brain activation maps during stimulation of the affected side limb in chronic lower back pain patients and normal controls. The areas indicated by the red arrows correspond to the primary motor cortex and supplementary motor area.

During the toe dorsiflexion task, the normal control group showed activation in the contralateral primary motor cortex and supplementary motor area, with a wide and concentrated range of strong activation (specific brain regions detailed in Figure 1 and Table 3). However, in the cLBP patient group, when the affected limb was activated, there was evident diffuse distribution and reduced intensity of activation in the primary motor cortex and supplementary motor area (specific brain regions detailed in Figure 2 and Table 3). This suggests compromised functionality of the motor central region during movement in the patient group.

**Table 3:** Brain activation regions during sensory stimulation and motor in chronic lower back pain patients.

Region of brain	Cluster size	MNI			T value	*p* value
		X	Y	Z		
Control group with sensory stimulation						
Postcentral_L	1,560	−39	−21	57	18.8285	*p* < 0.05
Supp_Motor_Area_L		−6	−12	54	11.9107	*p* < 0.05
SupraMarginal_L		−60	−24	18	8.1002	*p* < 0.05
Cerebelum_4_5_R	1,064	21	−51	−21	13.3143	*p* < 0.05
Cerebelum_Crus1_R		42	−48	−33	10.2417	*p* < 0.05
Precentral_R	1,030	63	3	27	12.2283	*p* < 0.05
Precentral_R		48	−15	54	12.0456	*p* < 0.05
Temporal_Mid_R		66	−51	3	11.1194	*p* < 0.05
Precentral_L	1,560	−39	−21	57	18.8285	*p* < 0.05
Supp_Motor_Area_L	1,560	−6	−12	54	11.9107	*p* < 0.05
cLBP group with sensory stimulation						
Paracentral_Lobule_L	74	−9	−21	78	5.3994	*p* < 0.05
Postcentral_L		−24	−33	66	3.4062	*p* < 0.05
Supp_Motor_Area_R	26	9	−12	75	4.4923	*p* < 0.05
Paracentral_Lobule_R	12	6	−30	75	4.2963	*p* < 0.05
SupraMarginal_R	114	63	−48	24	4.1727	*p* < 0.05
Parietal_Sup_L	24	−18	−45	66	3.6527	*p* < 0.05
Cerebelum_Crus1_L	83	−42	−78	−24	3.631	*p* < 0.05
Temporal_Mid_R	55	48	−69	−3	3.5918	*p* < 0.05
Cuneus_L	58	−3	−87	27	3.907	*p* < 0.05
Control Group with motor						
Frontal_Sup_2_L	1763	−27	0	72	22.9362	*p* < 0.05
Supp_Motor_Area_L	1763	−6	3	78	18.3433	*p* < 0.05
Precentral_L	1763	−42	−6	39	12.8644	*p* < 0.05
ParaHippocampal_L	205	−30	−42	−6	12.3126	*p* < 0.05
Lingual_L	205	−15	−57	−3	8.5865	*p* < 0.05
Frontal_Mid_2_L	164	−45	24	45	11.2989	*p* < 0.05
Angular_R	119	51	−48	27	11.2819	*p* < 0.05
Rolandic_Oper_R	138	51	−15	15	5.8061	*p* < 0.05
Frontal_Mid_2_R	52	36	3	60	9.6757	*p* < 0.05
Precuneus_R	296	15	−48	24	9.5364	*p* < 0.05
Cuneus_L	296	3	−72	24	6.0046	*p* < 0.05
Postcentral_R	89	60	−6	39	9.4283	*p* < 0.05
Lingual_R	216	24	−48	−3	8.8703	*p* < 0.05
Precentral_R	133	33	−27	63	7.166	*p* < 0.05
cLBP Group with motor						
Frontal_Mid_2_R	55	36	54	21	4.671	*p* < 0.05
Precentral_R	75	57	−9	42	4.58	*p* < 0.05
Postcentral_R	43	45	−30	63	4.457	*p* < 0.05
Precentral_R	50	51	9	36	3.945	*p* < 0.05
Frontal_Sup_2_R	23	24	21	39	3.631	*p* < 0.05
Frontal_Sup_2_L	37	−30	60	18	3.618	*p* < 0.05

Please consult the “Abbreviation for Brain Regions” section at the end of the paper for the complete names of brain regions.

### Resting-state functional MRI

In rs-fMRI studies, ALFF is an important metric used to calculate the intensity of local brain activity. Since the blood oxygen level dependent (BOLD) signal measured by fMRI is an indirect reflection of neural electrophysiological activity and the timescales of fMRI and disease-induced functional changes differ significantly, ALFF is thought to mainly reflect the intensity of neural electrophysiological activity within the low-frequency range.

**Figure 3:**
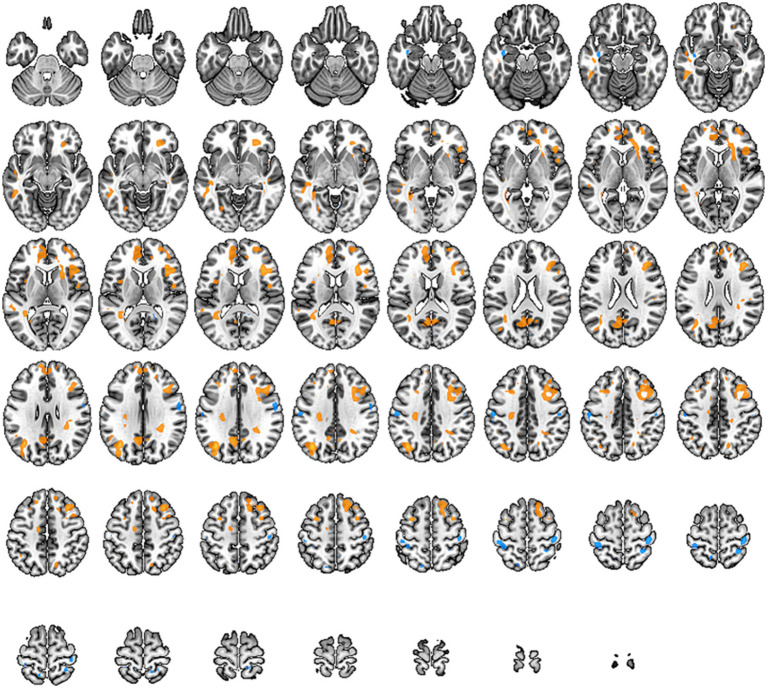
Brain regions with altered Amplitude of Low-Frequency Fluctuations (ALFF) across the whole brain. Warm tones represent an increase, while cool tones represent a decrease.

In this study, we observed changes in the ALFF values of brain regions responsible for cognitive function in the frontal lobe, the primary sensory areas in the sensorimotor network, and the pain-related regions such as the posterior cingulate cortex and the precentral gyrus. These alterations indicate changes in pain-related networks and sensory-motor networks in individuals with low back pain. These changes show a high correlation with the symptoms of pain and restricted movement function (specific brain regions detailed in Figure 3 and Table 4).

**Table 4:** Brain regions with altered amplitude of low-frequency fluctuations (ALFF) across the whole brain.

Region of brain	Cluster size	MNI			T value	*p* value
		X	Y	Z		
Occipital_Mid_L	50	−36	−66	33	8.1002	*p* < 0.05
Frontal_Inf_Tri_R	38	36	27	12	4.349	*p* < 0.05
Frontal_Inf_Tri_R	15	39	30	24	3.402	*p* < 0.05
Precuneus_L	22	−9	−60	33	2.976	*p* < 0.05
Frontal_Mid_2_R	20	36	24	48	2.936	*p* < 0.05
Postcentral_R	635	33	−45	63	2.882	*p* < 0.05
Postcentral_R		57	0	33	−4.502	*p* < 0.05
Postcentral_R		54	−18	42	−4.138	*p* < 0.05
Postcentral_L	974	−54	−15	39	−3.347	*p* < 0.05
Postcentral_L		−39	−39	63	−3.947	*p* < 0.05
Parietal_Sup_L		−15	−69	57	−3.822	*p* < 0.05
Cuneus_R	367	9	−81	21	−3.802	*p* < 0.05
Calcarine_R		21	−54	3	−3.609	*p* < 0.05
Cuneus_L		−9	−90	27	−3.516	*p* < 0.05
Heschl_L	32	−45	−21	9	−3.095	*p* < 0.05
Fusiform_R	39	39	−6	−30	−2.949	*p* < 0.05
Parietal_Sup_R	23	21	−66	57	−2.887	*p* < 0.05
Cingulate_Mid_R	15	6	12	33	−2.749	*p* < 0.05
Paracentral_Lobule_L	21	−3	−36	54	−2.707	*p* < 0.05
SupraMarginal_L	16	−63	−36	24	−2.673	*p* < 0.05

Please consult the “Abbreviation for Brain Regions” section at the end of the paper for the complete names of brain regions.

Regional Homogeneity, known as ReHo, is a measure of functional integration within brain regions and can be used to study local connectivity in specific areas. It analyzes the relationship between a particular voxel and its neighboring voxels, using the Kendall’s coefficient of concordance to detect if their activities are correlated. Increased ReHo values indicate enhanced consistency of spontaneous neural activity in local brain regions, while decreased values suggest reduced consistency.

**Figure 4:**
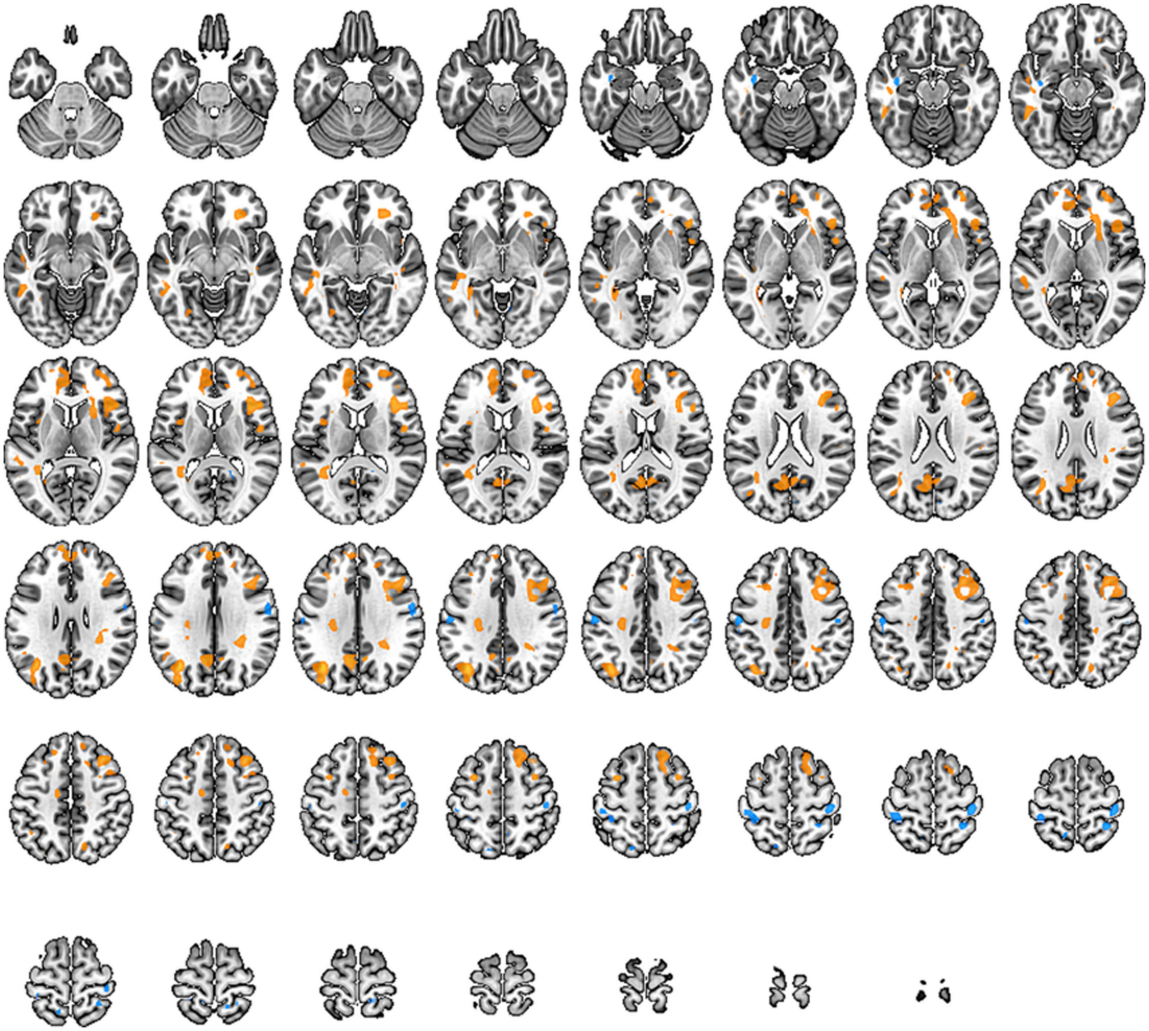
Brain regions with altered Regional Homogeneity (Reho) across the whole brain. Warm tones represent an increase, while cool tones represent a decrease.

In our study, individuals with low back pain showed significant changes in ReHo values in regions such as the frontal lobe, precentral gyrus, anterior cingulate cortex, hippocampus, putamen, primary motor area, primary sensory area, thalamus, and parahippocampal gyrus (specific brain regions detailed in Figure 4 and Table 5). The alterations in the frontal lobe and parahippocampal gyrus may be related to emotional and cognitive states, while changes in the primary sensory area, thalamus, putamen, and anterior cingulate cortex could be related to pain states. Changes in the primary motor area and putamen might be correlated with partial restrictions in movement function for these patients.

**Table 5:** Brain regions with altered regional homogeneity (Reho) across the whole brain.

Region of brain	Cluster size	MNI			T value	*p* value
		X	Y	Z		
Frontal_Inf_Tri_R	23	51	21	15	5.36	*p* < 0.05
Frontal_Mid_2_R	55	36	24	51	4.25	*p* < 0.05
Precuneus_R	17	12	−72	48	3.992	*p* < 0.05
Frontal_Sup_2_R	72	18	36	57	3.61	*p* < 0.05
		21	12	66	2.917	*p* < 0.05
Frontal_Mid_2_R	24	36	6	60	3.483	*p* < 0.05
Precuneus_R	11	6	−60	33	3.388	*p* < 0.05
Cingulate_Ant_R	37	15	42	15	3.127	*p* < 0.05
Hippocampus_R	15	24	−12	−15	3.123	*p* < 0.05
Temporal_Inf_L	25	−57	−33	−24	2.926	*p* < 0.05
Rectus_L	15	−3	51	−18	2.914	*p* < 0.05
Putamen_R	23	33	−3	−3	2.752	*p* < 0.05
Precuneus_L	11	−9	−57	30	2.62	*p* < 0.05
Caudate_L	10	−15	15	15	2.256	*p* < 0.05
Postcentral_L	835	−57	−21	30	−6.101	*p* < 0.05
Precentral_L		−42	−6	45	−4.892	*p* < 0.05
		−33	−21	63	−3.669	*p* < 0.05
Fusiform_R	37	36	−30	−27	−4.27	*p* < 0.05
Postcentral_R	182	57	−3	30	−4.199	*p* < 0.05
Precentral_R		48	−3	48	−3.397	*p* < 0.05
Postcentral_R		48	−27	54	−2.572	*p* < 0.05
Thalamus_R	166	6	−9	3	−4.083	*p* < 0.05
Thalamus_L		−12	−9	12	−3.284	*p* < 0.05
Fusiform_L	134	−36	−30	−27	−3.857	*p* < 0.05
ParaHippocampal_L		−27	0	−33	−3.291	*p* < 0.05

Please consult the “Abbreviation for Brain Regions” section at the end of the paper for the complete names of brain regions.

DC is a functional metric based on graph theory, used to measure the centrality of brain regions within a network. The centrality of an individual brain region (node) is equal to the number of edges connected to that node, which represents the node’s degree or number of neighbors. Therefore, the distribution of DC describes the centrality of all nodes within the network. In functional connectivity, high DC indicates greater importance within the network and reflects the node’s significance.

**Figure 5:**
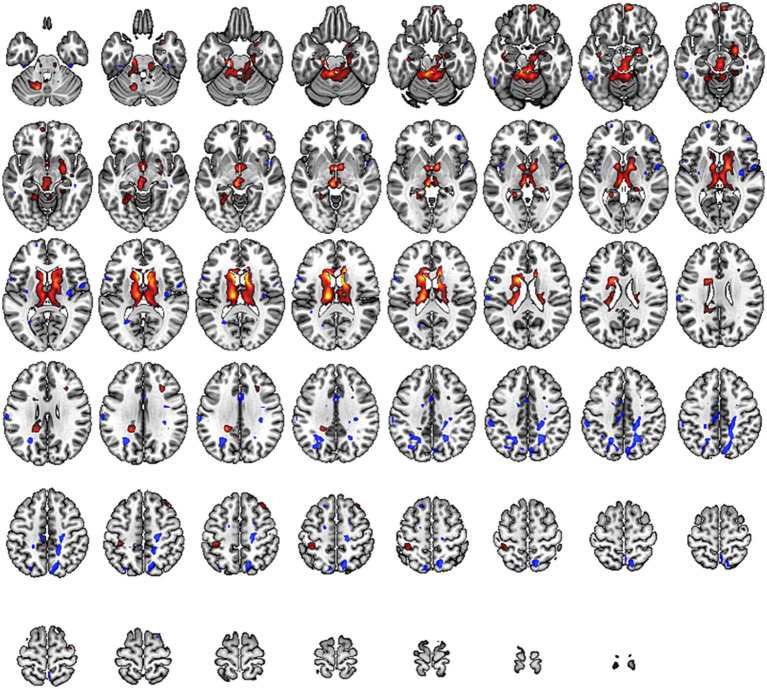
Brain regions with altered Degree Centrality (DC) across the whole brain. Warm tones represent an increase, while cool tones represent a decrease.

In our study, we observed a significant increase in DC values within the sensory-motor network located beneath the cortex, including the putamen, thalamus, anterior cingulate cortex, and paracentral lobule (specific brain regions detailed in Figure 5 and Table 6). This suggests that compression of peripheral nerves leads to abnormal peripheral signal transmission. Consequently, the brain compensates for impaired peripheral nerve input by enhancing stronger multisensory integration and motor feedback within the basal ganglia region. These findings imply adaptive changes in the brain to counteract the abnormalities caused by cLBP.

**Table 6:** Brain regions with altered degree centrality (DC) across the whole brain.

Region of brain	Cluster size	MNI			T value	*p* value
		X	Y	Z		
Caudate_L	1,595	−9	12	15	5.466	*p* < 0.05
Thalamus_L	1,595	−12	−18	15	4.906	*p* < 0.05
Frontal_Med_Orb_R	82	9	66	−15	3.976	*p* < 0.05
OFCmed_L	82	−12	60	−18	2.652	*p* < 0.05
Cerebelum_Crus1_L	60	−15	−66	−30	3.968	*p* < 0.05
Cerebelum_6_L	60	−30	−42	−39	2.175	*p* < 0.05
Postcentral_L	83	−36	−39	57	2.839	*p* < 0.05
		−51	−21	57	2.44	*p* < 0.05
ParaHippocampal_R	25	27	6	−27	2.814	*p* < 0.05
Cingulate_Ant_L	19	−6	24	18	2.761	*p* < 0.05
Frontal_Sup_2_R	15	36	−9	69	2.725	*p* < 0.05
Cerebelum_4_5_L	19	−33	−30	−33	−3.858	*p*< 0.05
Frontal_Inf_Oper_L	37	−60	12	18	−3.763	*p* < 0.05
Postcentral_L	75	−66	−21	24	−3.759	*p* < 0.05
Parietal_Inf_L	75	−60	−30	42	−2.862	*p* < 0.05
Precuneus_R	191	6	−81	45	−3.371	*p* < 0.05
		12	−66	57	−2.841	*p* < 0.05
Parietal_Sup_L	134	−18	−60	42	−3.384	*p* < 0.05
		−30	−75	51	−2.112	*p* < 0.05
Rolandic_Oper_R	101	57	0	6	−3.211	*p* < 0.05
Putamen_R		33	−12	6	−2.657	*p* < 0.05
Cingulate_Mid_R	30	18	−39	42	−3.17	*p* < 0.05
Cingulate_Mid_L	33	−6	−24	45	−2.695	*p* < 0.05
Frontal_Mid_2_R	33	45	45	3	−2.669	*p* < 0.05
Cingulate_Mid_L	48	0	6	36	−2.638	*p* < 0.05
Frontal_Sup_2_L	13	−18	27	57	−2.394	*p* < 0.05
Temporal_Mid_L	38	−54	−42	−12	−2.31	*p* < 0.05

Please consult the “Abbreviation for Brain Regions” section at the end of the paper for the complete names of brain regions.

Functional network connectivity is a crucial indicator used in rs-fMRI to describe the information transmission between different brain regions. It calculates the correlation of activity between brain regions that are spatially distant, characterizing the functional state of the brain’s internal networks.

**Figure 6:**
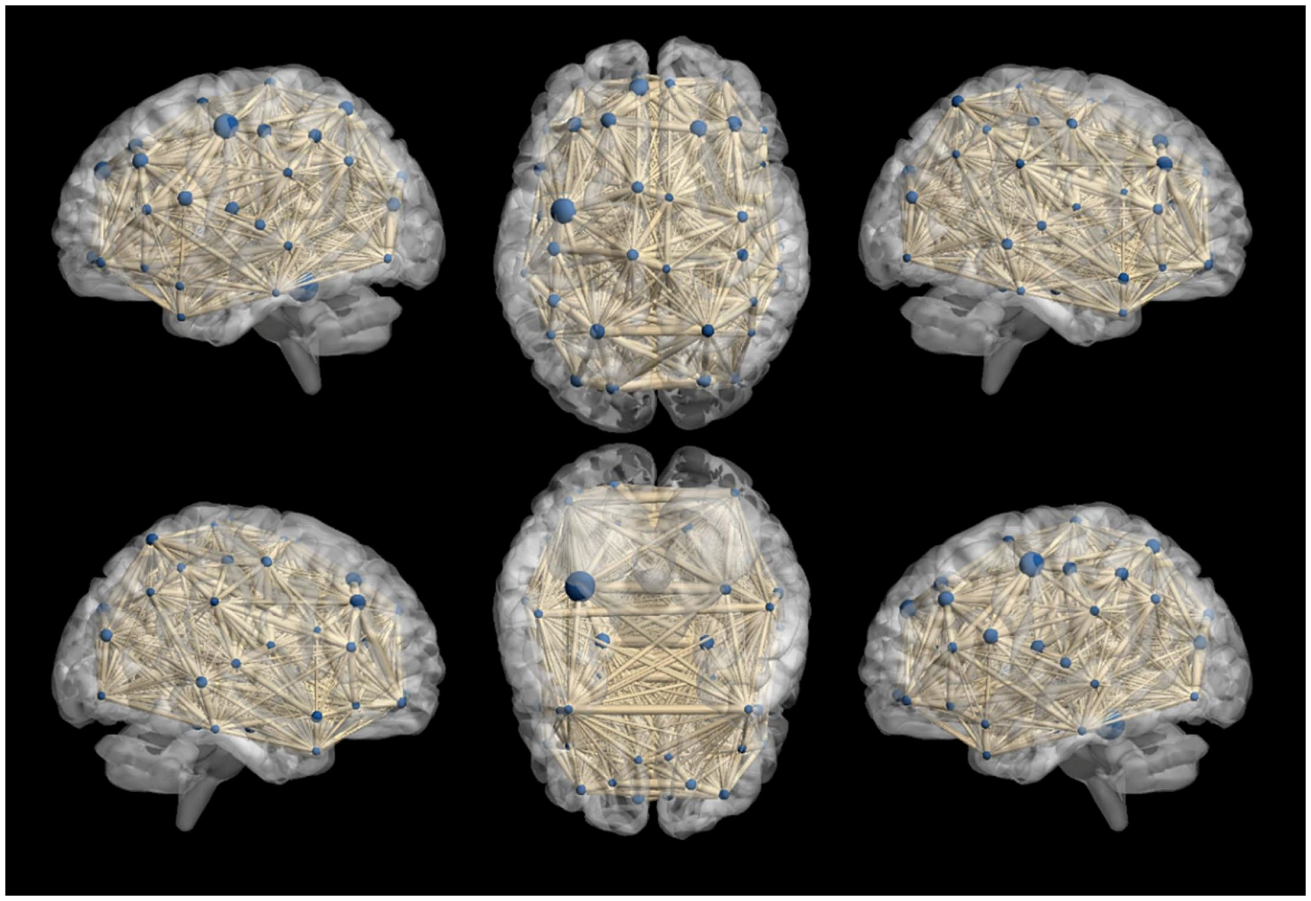
Whole-brain functional connectivity reconstruction in the cLBP group using the Automated Anatomical Labeling (AAL) template. The blue spheres represent different brain regions, and the thickness of the connecting sticks between the spheres represents the strength of the connections.

**Figure 7:**
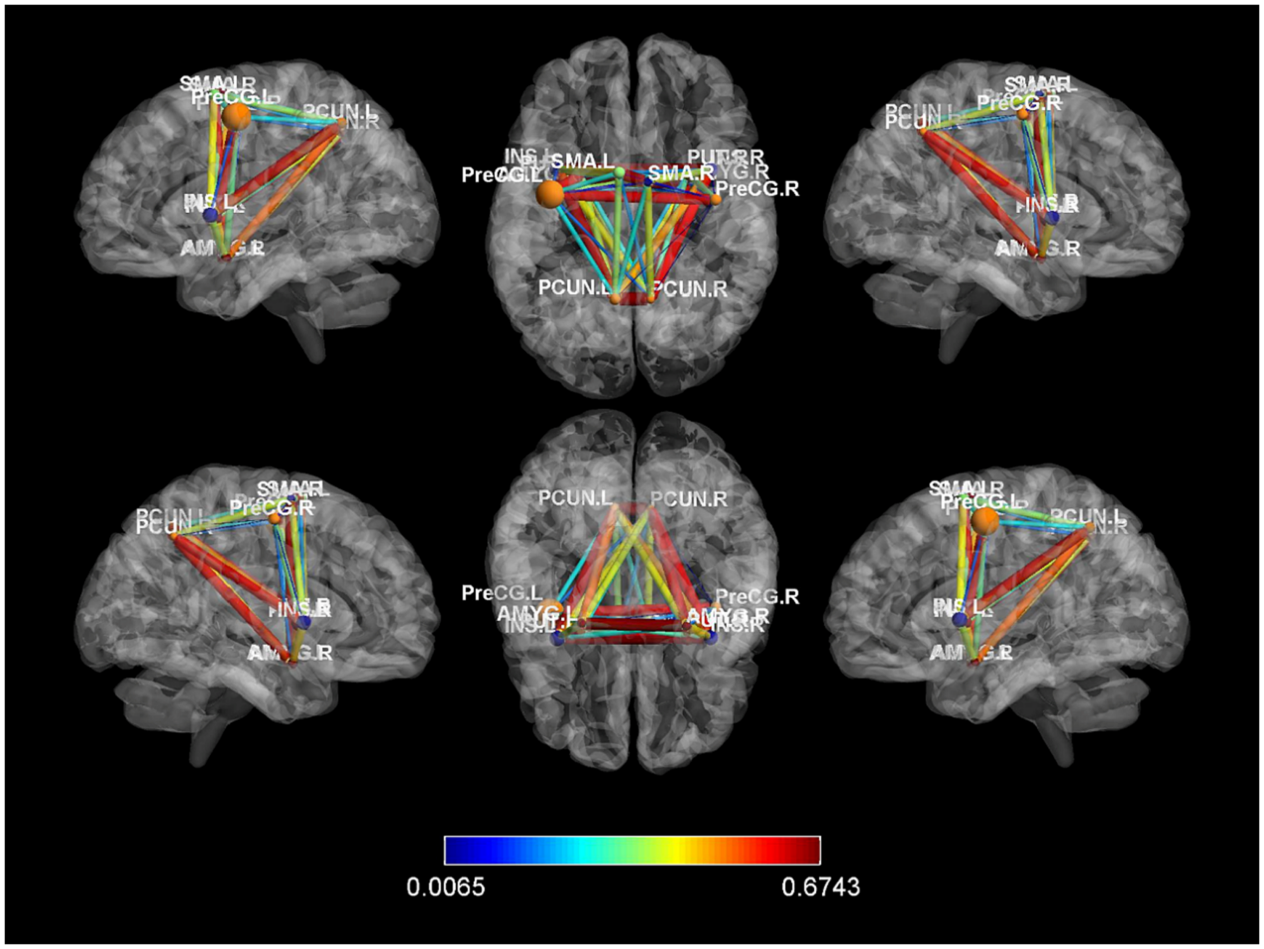
Differences in functional connectivity between chronic lower back pain group and normal control group. PreCG, Precentral gyrus; PCUN, Precuneus; AMYG, Amygdala; SMA, Supplementary motor area; L, left; R, right.

In our study, we constructed whole-brain network graphs using the AAL2 brain functional template and conducted comparative analysis of these networks between the two groups of participants. We found changes in functional connectivity within the sensory-motor network. Specifically, alterations were observed in connections from the affected side’s supplementary motor area to the primary motor area, from the primary sensory area to the primary motor area, and from subcortical nuclei (putamen, thalamus) to the primary motor area (specific brain regions detailed in Figures 6, 7). These changes are directly related to cLBP and the brain’s functional remodeling in patients. These findings provide important clues to better understand the neural mechanisms and brain adaptability associated with cLBP.

## Discussion

Lumbar pain is a complex disease, and its causes involve various aspects such as psychological, social, biomechanical, comorbidities, and pain processing mechanisms, resulting in significant variability in its etiology (Hartvigsen et al., 2018). For most lumbar pain patients, the exact cause or source of injury cannot be definitively determined, so these patients are generally referred to as non-specific low back pain (Maher et al., 2017). Only a small percentage of people can trace back to specific pathological causes, such as vertebral fractures, malignancies, rheumatoid arthritis, or infections, among others. Clinical studies have shown that low back pain often recurs multiple times, causing great suffering for patients and increasing the difficulty of clinical treatment (Maher et al., 2017). In recent years, the incidence of LDH has been gradually increasing. However, many patients lack awareness of the condition, preventing them from receiving appropriate treatment and resulting in suboptimal outcomes with conservative therapies (Knezevic et al., 2021). If timely surgical intervention is not possible, it further develops into chronic pain. Gaining a thorough understanding of the mechanisms underlying chronic pain is beneficial for providing targeted treatment references in clinical practice.

This study employed a multidimensional brain functional structural level using the brain-region-circuit network to thoroughly investigate the impact of chronic lower back pain on brain plasticity. In the task-based study, it was observed that chronic lower back pain patients exhibited decreased and diffuse activation in the somatosensory and motor cortices during tasks involving ankle dorsiflexion and sensory stimulation, suggesting impaired excitability in the sensory-motor central functions.

Resting-state fMRI, compared to other methods like positron emission tomography (PET), offers excellent spatial resolution and relatively good temporal resolution. This technique allows for safe and non-invasive visualization of human brain activity and has been widely used in pain research (Mehta et al., 2020). ALFF reflects the spontaneous activity of local neurons in the brain and serves as an index of endogenous neural physiological processes (Zou et al., 2008). Its greatest advantage lies in its ability to reveal the strength and characteristics of spontaneous neural activity in key brain regions. Some studies have linked ALFF with subjects’ cerebral blood flow and task-induced activations. Research indicates that the Default Mode Network (DMN) is one of the major networks affected by chronic pain (Mansour et al., 2013), and its functional disruptions may be associated with cognitive and behavioral impairments in chronic pain patients.

Our study reveals that at the brain-region level in the ALFF study, changes in ALFF values were observed in brain regions responsible for cognitive function, such as the frontal lobe, as well as in primary sensory areas and pain-related regions within the somatosensory-motor network, including the postcentral gyrus and the cingulate gyrus. These changes reflect adjustments in pain-related and sensory-motor network functions in patients, which are significantly correlated with pain and restricted movement symptoms.

Taking Zhang et al.’s study as an example, they used the ALFF method to investigate brain functional changes in chronic lower back pain patients (Zhang et al., 2019). The results showed increased ALFF values in the central anterior cingulate cortex, paracentral lobule, supplementary motor area, and anterior cingulate cortex in the patient group. This suggests that the changes in ALFF values in these regions may be related to the neuropathology of chronic lower back pain. Additionally, the study found that when patients’ spontaneous lower back pain intensity increased, the ALFF values of the insula, amygdala, hippocampus/parahippocampus, and thalamus increased, while the ALFF values in related brain regions within the DMN decreased. This indicates that these regions are more sensitive to experimental-induced changes in spontaneous lower back pain intensity.

Reho was first introduced by Yu-feng Zang and colleagues, and it is used to assess the temporal consistency of blood oxygen level-dependent signals in local brain tissue during resting state (Lou et al., 2020). Although Reho cannot directly measure the intensity of local neuron activity, it can reflect the synchronization of neuron activity in local brain regions, making it a reliable and effective indicator. Currently, the Reho method has been applied in related fields such as idiopathic trigeminal neuralgia and discogenic lumbar radicular pain.

Our study reveals that at the brain-region level in the ReHo study, significant changes in Reho values were found in regions including the frontal lobe, anterior cingulate cortex, supplementary motor area, hippocampus, putamen, primary motor cortex, primary somatosensory cortex, thalamus, and hippocampal parahippocampal gyrus. Alterations in the frontal lobe and hippocampal parahippocampal gyrus might be related to emotional and cognitive states, whereas changes in the primary somatosensory cortex, thalamus, putamen, and anterior cingulate cortex were associated with pain states. Changes in the primary motor cortex and putamen were related to limited motor functions.

In the study by other researchers, the Reho analysis method was employed to investigate changes in resting-state brain activity in experimentally induced lower back pain subjects (Zhang et al., 2014). The study found that compared to the baseline state, the Reho values increased in several brain regions, including the anterior cingulate cortex, insular cortex, hippocampal gyrus, and posterior cerebellum, during experimentally induced lower back pain. Meanwhile, the Reho values decreased in multiple regions, including the left anterior cingulate cortex, primary somatosensory cortex, and hippocampal gyrus. These findings suggest that abnormal resting-state activity in certain brain regions may be associated with the occurrence of pain, and these changes may affect the identification, execution, memory, and emotional processing of acute lower back pain.

FC primarily assesses the temporal correlation between brain regions to reflect the efficiency of connections and coordinated interactions between anatomically separated brain regions. Pei Yixiu et al. employed ROI analysis technique and found abnormal functional connectivity in the primary somatosensory cortex of patients with discogenic lumbar radicular pain (Pei et al., 2020). Moreover, in our network-level exploration of whole-brain functional connectivity, changes were observed in the sensory-motor network, including altered functional connectivity from the affected-side supplementary motor area to the primary motor area, primary somatosensory area to the primary motor area, and subcortical nuclei (caudate nucleus, putamen) to the primary motor area. These changes are directly related to chronic lower back pain and the restructuring of brain functions.

Yu et al. conducted FC analysis with periaqueductal grey (PAG) as a seed point using ROI analysis technique (Yu et al., 2014). The results showed enhanced functional connectivity between the PAG and the anterior insula in chronic lower back pain patients. This abnormal FC was negatively correlated with pain duration. This suggests that patients with chronic lower back pain exhibit abnormal functional connectivity in the pain modulation network centered around the PAG, which could have significant implications for pain treatment. Additionally, further studies on other LDH patients reveal enhanced functional connectivity between the thalamus and dorsolateral prefrontal cortex (DLPFC). This further influences the relationship between chronic pain and depression. The results emphasize the potential crucial role of the thalamic pathway to the prefrontal cortex in regulating chronic pain and depression in the pathophysiology of LDH (Li et al., 2021).

Our study reveals that at the network level in the DC study, regions within the sensory-motor network’s subcortical areas, including the caudate nucleus, putamen, anterior insula, and supplementary motor area, exhibited increased node degree values. This could be attributed to peripheral nerve root compression leading to abnormal peripheral signal transmission, thus requiring the brain to enhance compensatory integration in the basal ganglia for movement feedback networks. Liu, Jing et al. have found that, compared to the control group, patients with lumbar disk herniation exhibit significantly longer characteristic path lengths in the brain network, as well as lower clustering coefficients, global efficiency, and local efficiency (Liu et al., 2018). In comparison to the healthy control group, individuals with low back pain often demonstrate an unstable and less efficient brain network.

Prior neuroimaging studies on brain microstructure have shown that gray matter volume in the prefrontal cortex of cLBP patients decreases (Luchtmann et al., 2014; Jenkins et al., 2022). The prefrontal cortex involves multiple brain regions related to pain processing, and these regions are extensively interconnected by fibers that can regulate pain through reward stimuli (Becker et al., 2017). Furthermore, multiple studies suggest functional abnormalities in various brain regions, including bilateral prefrontal regions, among chronic pain patients (Zhou et al., 2018; Vachon-Presseau et al., 2019). However, research on the brain functional effects of cLBP caused by LDH remains limited. Therefore, gaining a deeper understanding of the patterns of spontaneous brain neural activity and whole-brain functional connectivity in such patients can contribute to our comprehension of central pain modulation mechanisms in cLBP.

Currently, the effectiveness of conservative treatment for low back pain is often unsatisfactory, and there is significant variability in treatment outcomes among different patients. Opioid medications are commonly used to treat patients with low back pain, but some studies suggest that this may increase patients’ reliance on opioids and their risk of addiction (Deyo et al., 2015). Clinical research also shows that the dependency and addictive nature of opioids could impact the normal structure and physiological function of the brain. After conducting preliminary research on cLBP patients who had been taking opioids for a long period, Murray et al. found a significant reduction in the volume of the thalamus and thalamus nuclei and a general decrease in signal-to-noise ratio in cortical areas (Murray et al., 2021). These research findings suggest that long-term use of opioid medications may be related to structural and functional changes in the sensory-motor system of the brain in cLBP patients. Another study conducted by Lin et al. found that within a month of taking opioid medications, gray matter volume decreased in the amygdala and other brain regions associated with reward processing in low back pain patients, while gray matter volume increased in the anterior cingulate cortex (ACC) region (Lin et al., 2016). These research findings suggest that opioid medications may influence the brain structure and function of patients. Consequently, investigations into brain remodeling among individuals with cLBP have emerged as a frontier area of study. Gaining insights into the alterations in brain due to pain, along with its multidimensional effects on patients’ sensations, cognition, emotions, and beyond, holds profound clinical significance.

This study offers a multi-layered analysis spanning brain regions, circuits, and networks. It sheds light on the diminished excitability of sensory-motor areas in patients with chronic lower back pain resulting from LDH during task states. Additionally, the research uncovers heightened activity in multiple nuclei within the sensory-motor network and changes in functional connectivity between various brain regions during resting states. These findings may significantly contribute to the understanding of neurobiological mechanisms involved in chronic lower back pain, providing valuable insights for the exploration of future treatment approaches and laying the groundwork for enhancing clinical efficacy in symptom management.

This study also has several limitations. Firstly, the sample size is relatively small, potentially impacting statistical analyses and the comprehensive interpretation of results. Secondly, being a single-time-point investigation, this study lacks follow-up assessments on brain function after treatment, thus hindering the evaluation of dynamic and temporal changes in brain activity. This aspect requires further refinement in future longitudinal studies. Thirdly, the enrolled patients in this study do not represent the entire age spectrum, which may limit the generalizability of the findings to other populations. Future research should involve a broader age range to yield more inclusive results.

## Data availability statement

The raw data supporting the conclusions of this article will be made available by the authors, without undue reservation.

## Ethics statement

The studies involving humans were approved by the study was conducted in accordance with the Decla-ration of Helsinki and approved by the Ethics Committee of the Second People’s Hospital of Changshu (No. 2021-KYW-035). The studies were conducted in accordance with the local legislation and institutional requirements. The participants provided their written informed consent to participate in this study.

## Author contributions

Y-DM: Writing – original draft. HG: Writing – original draft, Methodology. W-FC: Writing – original draft, Formal analysis. WZ: Writing – original draft, Formal analysis. CG: Writing – original draft, Validation. J-PZ: Writing – review & editing. J-MT: Writing – review & editing. X-YH: Writing – review & editing, Conceptualization.

## Footnotes

^1^ www.fil.ion.ucl.ac.uk/spm

## Glossary

Abbreviation for brain regions (Rolls et al., 2020).

Angular_R: Right Angular gyrus
Calcarine_R: Right Caudate nucleus
Caudate_L: Left Caudate nucleus
Cerebelum_4_5_L: Left Lobule IV V of cerebellar hemisphere
Cerebelum_4_5_R: Right Lobule IV V of cerebellar hemisphere
Cerebelum_6_L: Left Lobule VI of cerebellar hemisphere
Cerebelum_Crus1_L: Left Crus I of cerebellar hemisphere
Cerebelum_Crus1_R: Right Crus I of cerebellar hemisphere
Cingulate_Ant_L: Left Anterior cingulate & paracingulate gyri
Cingulate_Ant_R: Right Anterior cingulate & paracingulate gyri
Cingulate_Mid_L: Left Middle cingulate & paracingulate gyri
Cingulate_Mid_R: Right Middle cingulate & paracingulate gyri
Cuneus_L: Left Cuneus
Cuneus_R: Right Cuneus
Frontal_Inf_Oper_L: Left Inferior frontal gyrus, opercular part
Frontal_Inf_Tri_R: Right Inferior frontal gyrus, triangular part
Frontal_Med_Orb_R: Right Middle frontal gyrus, orbital part
Frontal_Mid_2_L: Left Middle frontal gyrus
Frontal_Mid_2_R: Right Middle frontal gyrus
Frontal_Sup_2_L: Left Superior frontal gyrus
Frontal_Sup_2_R: Right Superior frontal gyrus
Fusiform_L: Left Fusiform gyrus
Fusiform_R: Right Fusiform gyrus
Heschl_L: Left Heschl gyrus
Hippocampus_R: Right Hippocampus
Lingual_L: Left Lingual gyrus
Lingual_R: Right Lingual gyrus
Occipital_Mid_L: Left Middle occipital gyrus
OFCmed_L: Left Medial orbitofrontal cortex
Paracentral_Lobule_L: Left Paracentral lobule
Paracentral_Lobule_R: Right Paracentral lobule
ParaHippocampal_L: Left Parahippocampal gyrus
ParaHippocampal_R: Right Parahippocampal gyrus
Parietal_Inf_L: Left Inferior parietal gyrus, excluding supramarginal and angular gyri
Parietal_Sup_L: Left Superior parietal gyrus
Parietal_Sup_R: Right Superior parietal gyrus
Postcentral_L: Left Postcentral gyrus
Postcentral_R: Right Postcentral gyrus
Precentral_L: Left Precental gyrus
Precentral_R: Right Precental gyrus
Precuneus_L: Left Precuneus
Precuneus_R: Right Precuneus
Putamen_R: Right Lenticular nucleus, putamen
Rectus_L: Left Gyrus rectus
Rolandic_Oper_R: Right Rolandic operculum
Supp_Motor_Area_L: Left Supplementary motor area
Supp_Motor_Area_R: Right Supplementary motor area
SupraMarginal_L: Left Supramarginal gyrus
SupraMarginal_R: Right Supramarginal gyrus
Temporal_Inf_L: Left Inferior temporal gyrus
Temporal_Mid_L: Left Middle temporal gyrus
Temporal_Mid_R: Right Middle temporal gyrus
Thalamus_L: Left Thalamus
Thalamus_R: Right Thalamus
